# Spatiotemporal analysis of dengue fever in Nepal from 2010 to 2014

**DOI:** 10.1186/s12889-016-3432-z

**Published:** 2016-08-22

**Authors:** Bipin Kumar Acharya, ChunXiang Cao, Tobia Lakes, Wei Chen, Shahid Naeem

**Affiliations:** 1 University of Chinese Academy of Sciences, No.19A Yuquan Road, Beijing, 100049 China; 2 Institute of Remote Sensing and Digital Earth, Chinese Academy of Sciences, No.9 Dengzhuang South Road, Haidian District, Beijing, 100094 China; 3 Department of Geography, Humboldt-Universität zu Berlin, Unter den, Linden, 6, 10099 Berlin Germany

**Keywords:** Dengue, Nepal, Crude incidence, Excess risk, Cluster

## Abstract

**Background:**

Due to recent emergence, dengue is becoming one of the major public health problems in Nepal. The numbers of reported dengue cases in general and the area with reported dengue cases are both continuously increasing in recent years. However, spatiotemporal patterns and clusters of dengue have not been investigated yet. This study aims to fill this gap by analyzing spatiotemporal patterns based on monthly surveillance data aggregated at district.

**Methods:**

Dengue cases from 2010 to 2014 at district level were collected from the Nepal government’s health and mapping agencies respectively. GeoDa software was used to map crude incidence, excess hazard and spatially smoothed incidence. Cluster analysis was performed in SaTScan software to explore spatiotemporal clusters of dengue during the above-mentioned time period.

**Results:**

Spatiotemporal distribution of dengue fever in Nepal from 2010 to 2014 was mapped at district level in terms of crude incidence, excess risk and spatially smoothed incidence. Results show that the distribution of dengue fever was not random but clustered in space and time. Chitwan district was identified as the most likely cluster and Jhapa district was the first secondary cluster in both spatial and spatiotemporal scan. July to September of 2010 was identified as a significant temporal cluster.

**Conclusion:**

This study assessed and mapped for the first time the spatiotemporal pattern of dengue fever in Nepal. Two districts namely Chitwan and Jhapa were found highly affected by dengue fever. The current study also demonstrated the importance of geospatial approach in epidemiological research. The initial result on dengue patterns and risk of this study may assist institutions and policy makers to develop better preventive strategies.

## Background

Dengue fever is a mosquito-borne viral disease which is transmitted from one person to another through bites of female *Aedes–spp.* mosquito [1]. It is one of the major public health problems for tropical and subtropical countries all over the world. Nearly one third of the world population lives in countries under the risk of dengue fever. Annual dengue infection was estimated around one hundred millions globally [2]. Dengue transmission has expanded in new geographic areas and the severity of infections has increased in areas where infection was already endemic [3]. Global burden of dengue has exceeded malaria and the problem is likely to be more severe in the future [4] due to climate change, increasing trend of urbanization and migration [5]. Despite such massive problems, there are no effective initiatives to prevent dengue and no medicine for causal treatment available yet [6]. Therefore understanding the dynamics of dengue transmission seems imperative to reduce the public health burden.

The *Aedes aegypti* mosquito which is the main vector of dengue, lives in urban habitats and breeds mostly in man-made containers [7, 8]. Unlike other mosquitoes it is a daytime feeder; its peak biting periods are early in the morning and in the evening before dusk [8]. Several factors determine occurrences and spread of dengue by affecting life cycle and behavior of the mosquito. Temperature and rainfall are the most significant factors for vectors development and dynamics [4, 9–13]. Very low temperature limits not only egg hatching and larval development process [14] but also extrinsic incubation period and viral development rate [15]. Freezing temperatures in higher altitudes destroys larvae and eggs of mosquitoes during winter time [16]. Adult mosquito survival rates are linked with lower temperature and higher humidity. Rainfall is a source of fresh water for mosquito breeding in water containers. However excessive rainfall is negatively associated with dengue by washing out the eggs [17, 18]. Further, high population density and low socioeconomic status are positively associated with dengue occurrence [17–19]. Due to variation in these factors, occurrence and spread of dengue fever also vary over space and time. To understand the variation of dengue fever, several studies have been carried out to explore the spatiotemporal pattern and risk factors of dengue fever in other areas of the world [16–19].

In Nepal, dengue is an emerging disease which was first reported in 2004 [20]. Since then, it has been spreading rapidly over wide geographical areas. The number of both confirmed dengue cases and dengue reported districts are continuously increasing. Now, dengue is firmly established in the tropical and subtropical plains of Nepal, the Terai, and is migrating upwards [21] posing significant challenges to the public health officials. Till 2014, dengue has been reported in 32 districts and confirmed dengue cases reached 2442 and 5 deaths toll [22]. These statistics are even believed to be underreported and prevalence of dengue is considered significantly high [23]. *Ae. aegpti*is now widely distributed in major cities of the Terai and also migrated up to 2000 m altitude in response to climate change [24] posing a high risk of outbreak even in major cities in the hill districts (e.g. Kathmandu and Pokhara) [25]. All four serotypes of dengue virus circulate in Nepal with the host, vectors and the environment [26] which further increase risk of dengue infection and outbreak in Nepal. To our best knowledge, there are not scientific studies to explore spatial epidemiology of dengue in Nepal. For the improvement of government efforts to control dengue such studies would be of utmost importance.

In recent years, GIS (Geographic Information Systems) and spatial statistics were frequently used to characterize spatiotemporal patterns of dengue and other infectious as well as non-infectious diseases [19, 27, 28]. Cheong et al. assessed spatiotemporal patterns of dengue in Malaysia combining the address and sub district levels [29]. Banu et al. studied 50 years (1955–2004) spatiotemporal trends of dengue transmission in the Asia-pacific region [30]. Similarly spatial analysis of dengue in Guangdong province, China was conducted for incidence data from 2001 to 2006 [31]. Most of the studies analyzing spatiotemporal patterns of dengue have used SaTScan and GeoDa public domain software [19, 27, 28, 31]. GeoDa software provides several ways to visualize and map distribution pattern of disease by correcting for spatial autocorrelation and spatial dependencies [32]. SaTScan software provides a powerful tool to detect, delineate, and validate disease clusters, risk population, and factors associated with them over space and time. Further SaTScan adjusts for confounding variables, and reduces pre-selection bias regarding the size and location of clusters. The current study aims to assess and map spatiotemporal patterns of reported dengue cases based on monthly surveillance data aggregated at 75 districts of Nepal.

## Methods

### Study area

**Fig. 1:**
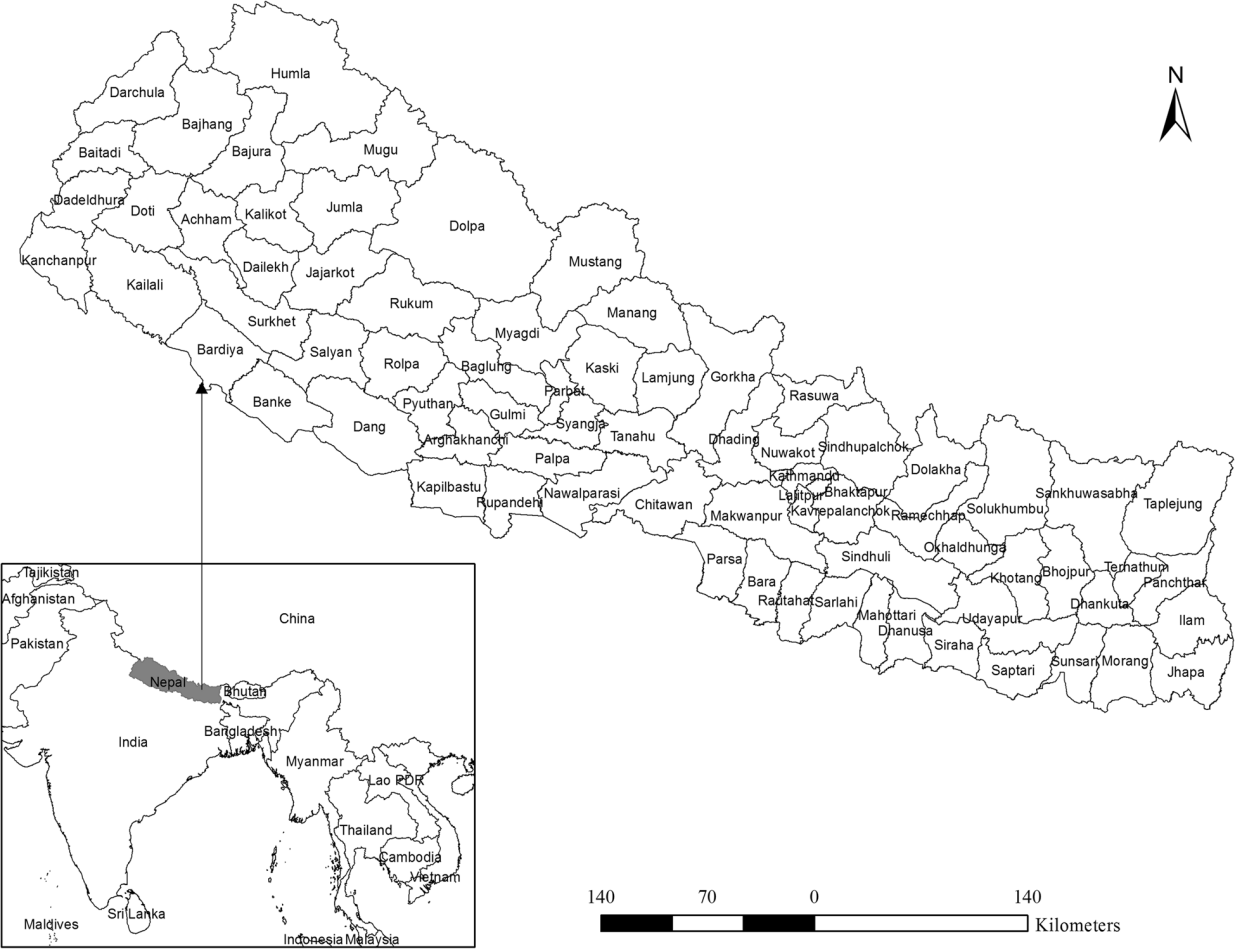
Location of the study area

The country Nepal is located roughly between 26° to 30°N in latitude and 80°to 88°E in longitude. Land topography is diverse with remarkable differences in elevation ranging from 60 m in southern lowland to 8840 m of the mighty Himalayas in the north. Administratively, Nepal is divided into 5 development regions, 14 zones, and 75 districts. In this study, the analysis was performed on the level of the 75 districts as shown in Fig. 1. Broadly, Nepal lies in a subtropical monsoon climate zone characterized by large seasonal variation in rainfall, temperature and humidity. Micro climatic variation is also prominent due to variation in altitude and topography. Summer and winter are the two major seasons. Summer is normally hot and humid, while conversely, winter is cold and dry. About 80 % of annual rainfall occurs during summer through monsoon. Mean annual rainfall generally decreases from east to west and from north to south and mean annual temperatures also follows approximately the same pattern.

### Data source

In this study, dengue incidence data was acquired from Epidemiology and Disease Control Division (EDCD) of Department of Health Services (DOHs). DOHs is, under the Ministry of Health and Population, responsible for collecting, processing and publishing disease data including dengue in Nepal. Disease data in Nepal are reported to the EDCD which is the Nepal government’s authority for the prevention and control of infectious disease. Disease data are usually reported on a weekly basis but reported dailyduring outbreak. For this study dengue data was available from 2010 to 2014. During the study period 2343 dengue cases were reported to EDCD based on either Immunoglobulin M (IgM) tests or Polymerase Chain Reaction (PCR) tests.

The district boundary map and the population data used in the study were obtained from Department of Survey, Government of Nepal, and the National Censes Report-2011, published by Central Bureau of Statistics (CBS) respectively.

### GIS mapping and smoothing

We used choropleth mapping technique to visualize the dengue count data for the 5 years at district level. To alleviate variations on dengue incidence for small population numbers and areas, we calculated annual rates of dengue from the count data. For this, we first computed the mean annual incidence rate per 100,000 population for each district by summing all the cases occurring each year in each district and dividing them by the corresponding district population. In the second step we averaged annual incidence rate for 5 years resulting in an averaged mean annual incidence rate of dengue per 100,000 populations per district (MAI _dist (i)_).

$$ MA{I}_{dist(i)}=\frac{1}{5}{\displaystyle \sum_{y2010}^{y2014}}\left[\left(\frac{DFcas{e}_{dist(i) year\left(\ y\right)}}{Populatio{n}_{dist(i) year(y)}}\right)*1000,000\right] $$

Where: *DF cases* are the dengue fever cases reported from the district (i) each year (y) from 2010–2014 and the *population is* population reported in 2011 census.

Following Fang et al., we computed a 5 years annual raw rate map [27]. This map was subject to spatial autocorrelation and therefore cannot provide real distribution information. Therefore, we further processed 5 years averaged incidence rate to produce spatially smoothed dengue distribution map through correction of spatial autocorrelation. To do this, we used the empirical Bayes approach [33] available in GeoDa. We first created a spatial weight file in GeoDa that contain s neighbored structure using the K-nearest neighborhood criteria (four districts in our case) which was later loaded to make spatially smoothed distribution maps. To assess the risk of dengue, an excess hazard map was computed. The excess hazard represents the ratio of observed incidence at each district over the average incidence of all endemic areas [34]. In the excess hazard map, value one is usually determined as a cut-off value whereas below one indicates lower incidence than expected and above it indicates incidence higher than expected. All GIS mapping and smoothing works were implemented in GeoDa, 1.6.7 software.

### Spatiotemporal cluster analysis

SaTScan software version 9.4.2 developed by Kuldlorff [35, 36] was used to detect and evaluate dengue clusters. All three scanning methods (purely spatial, purely temporal and spatiotemporal) were employed to assess the geographical areas with highest dengue risk neglecting the temporal dimension, to find highest risk period neglecting the space dimension and to locate space-time outbreak addressing the effect of purely spatial and purely temporal variation in the incidence data. Poisson-based model was employed in all three analyses.

SaTscan scans gradually across time and/or space to identify possible clusters by comparing the number of observed incidences and expected incidences (assuming random distribution) inside the window at each location. Scanning window is a time interval for purely temporal scan, a circle or ellipse in spatial scan and a cylinder in space-time scan where base of a cylinder represents space dimension and height represents the temporal dimension. The null hypothesis is that the risk of dengue incidence is equal throughout the study area while the alternative hypothesis is that the risk of dengue is different inside and outside of at least one circle or cylinder. The area of circle or cylinder varies from zero to the maximum specified cluster size of the total cases. In this way, the entire study area (space) or time is covered with varying size of circle or cylinder. Only clusters with significant levels with cut-off values such as 0.05, 0.01 and 0.001 after Monte Carlo simulation repeated e.g. 999 are reported. The cluster with the maximum log likelihood ratio is taken as the most likely cluster, i.e. the cluster least likely to be due to chance. The log likelihood ratio in Possion distribution is computed as:

$$ LIR=\left(\frac{C}{E(c)}\right)c\cdot \left(\frac{C-c}{C-E(c)}\right)c \cdot I\left(\right) $$

- Where:
- LIR = Log Likelihood Ratio
- C = total number of cases
- c = observed number of cases within the window
- E[c] = covariate adjusted expected number of cases within the window under the null hypothesis,
- I() = indicator function

For purely spatial and space-time analyses, SaTScan also identifies secondary clusters in the data in addition to the most likely cluster, and orders them according to their likelihood ratio test (LLR) statistic. SaTScan reports both geographically overlapping and non-overlapping secondary clusters. Due to the high log likelihood values with the most likely cluster, these clusters provide little additional information. However, non-overlapping secondary clusters are considered significant.

The maximum cluster size was set to 50 % of the population at risk for spatial scan; to account for differences in population density [37] and a non-overlapping secondary cluster was set to be reported [31, 37]. In temporal scan analysis, a value of 6 months was chosen for maximum temporal window size to capture seasonality in dengue incidence. In the space- time scan, purely spatial and purely temporal window parameters were taken. We chose high rates option in the scan for areas option to account for clusters.

## Results

### Spatial and temporal distribution of dengue in Nepal

**Fig. 2:**
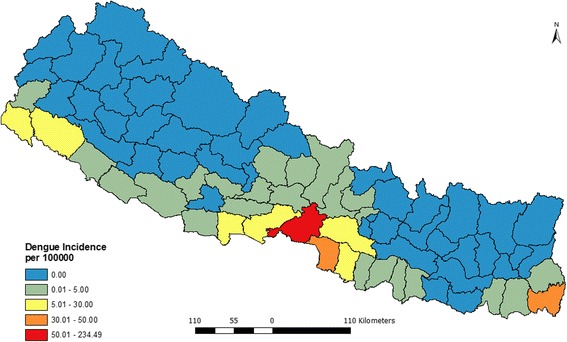
Annualized average incidence of dengue fever in Nepal (2010–2014)

A total of 2343 dengue cases were reported in Nepal from 2010 to 2014. Five years annual average incidence ranges from 0 to 234 per 100,000 populations (Fig. 2). Out of 75 districts, 43 were found non-endemic with zero incidences whereas 32 were found endemic to the disease. Among the 32 districts, 23 were low endemic (< 5 incidences), 6 were medium (5–30 incidences) and two were found highly endemic (30–50 incidences). With more than 50 incidences Chitwan district was found the most endemic among the districts under consideration where 234 people out of 100,000 were reported infected with dengue virus during the 5 year period.

**Fig. 3:**
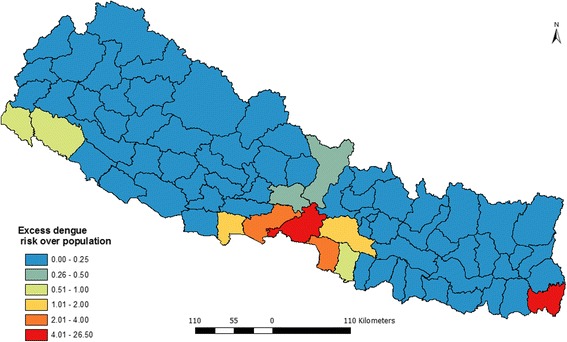
Excess hazard map of dengue fever in Nepal (2010–2014)

Figure 3 presents the distribution of excess risk of dengue in Nepal over 5 years (2010–2014). The figure shows that only 6 districts had the excess risk higher than expected while the 64 districts had excess risk lower than expected.

The intensity of risk is labeled using bipolar graduate symbol where red side shows excess risk higher than expected while the blue shows excess risks less than expected. Among the 6 districts withexcess risk higher than expected, Chitwan and Jhapa had highest excess hazard while the Makawanpur and Rupendehi had the lowest excess risk.

**Fig. 4:**
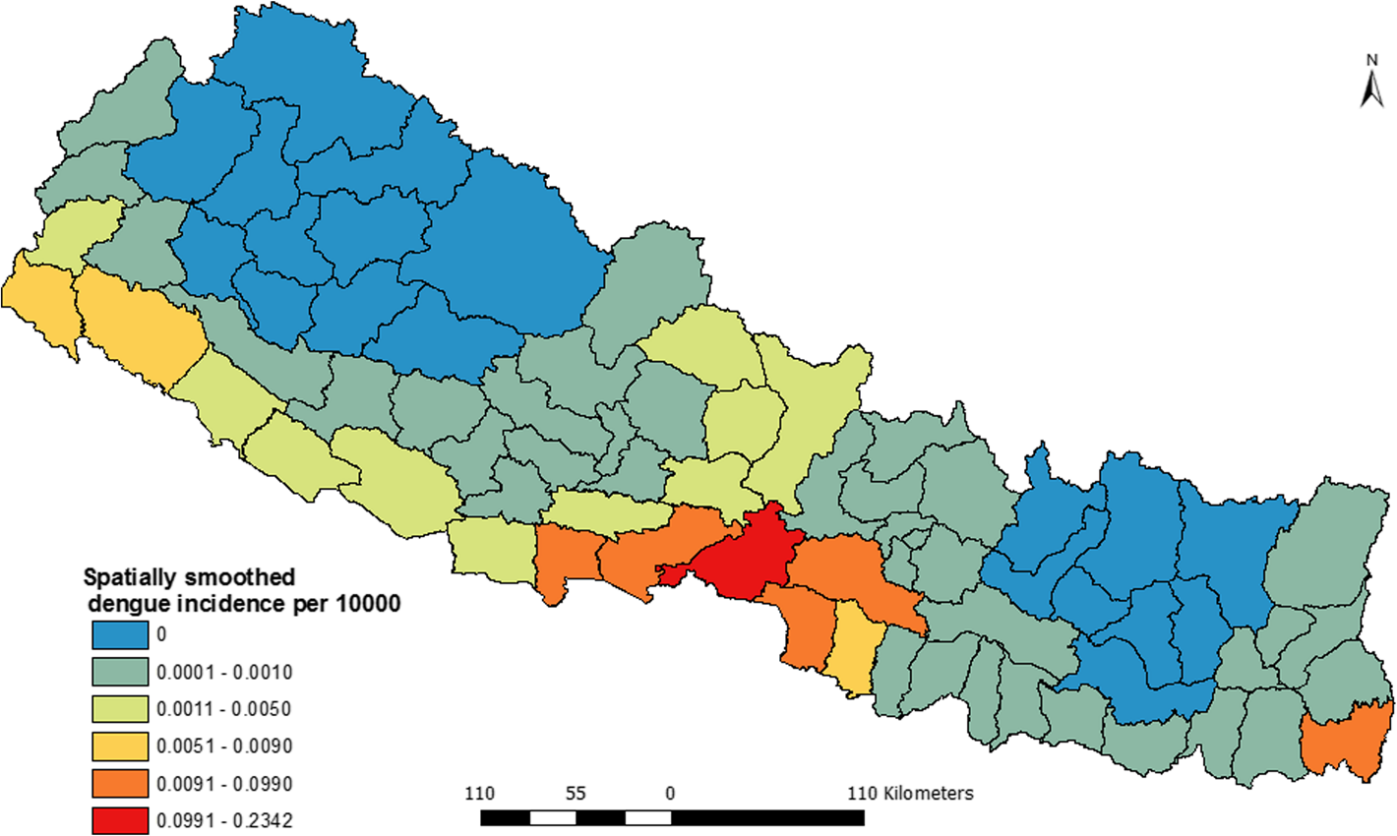
Spatially smoothed dengue incidence map of Nepal (2010–2014)

As shown in the Fig. 4, spatial variation of dengue incidences was corrected using K-nearest neighbor criteria based on the empirical bias approach. Due to implementation of smoothing, there is no spatial autocorrelation. Therefore, this map presents a better pattern of dengue incidence and shows clearly where dengue incidence was most severe. The map also shows that Chitwan and the adjacent four districts; Nawalparasi, Parsa, Rupendehi and Makawanpur are dengue prone zones in Nepal. Moreover Jhapa district is also an equally highly dengue vulnerable district. Besides these six, the districts from far western region (mainly Kailali and Kanchanpur) were found under high risk of dengue.

**Fig. 5:**
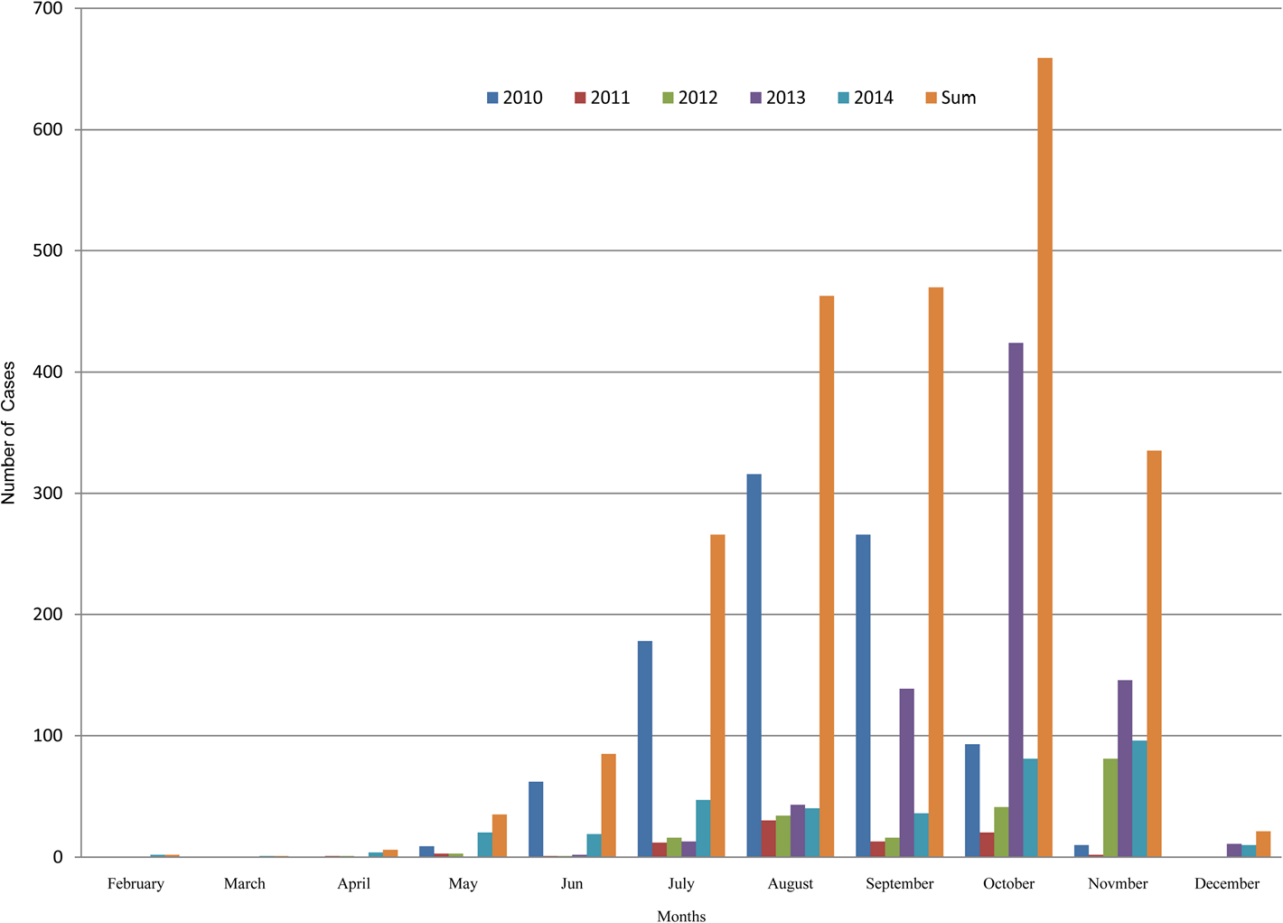
Monthly distribution of dengue fever in Nepal (2010–2014)

Dengue fever is highly seasonal in Nepal and follows the patterns of monsoon rainfall. Dengue cases start to appear with onset of monsoon and reach the highest peak in the following months. Normally, monsoon starts in early June and last for next 3 months. Nepal receives about 80 percentage of annual rainfall during this period. Figure 5 shows that August was the peak month of dengue outbreak in 2010 and the peak month of 2013 outbreak was October. October was also the peak for the sum cases of 2010–2014.

### Distribution of dengue clusters

#### Spatial clusters

**Fig. 6:**
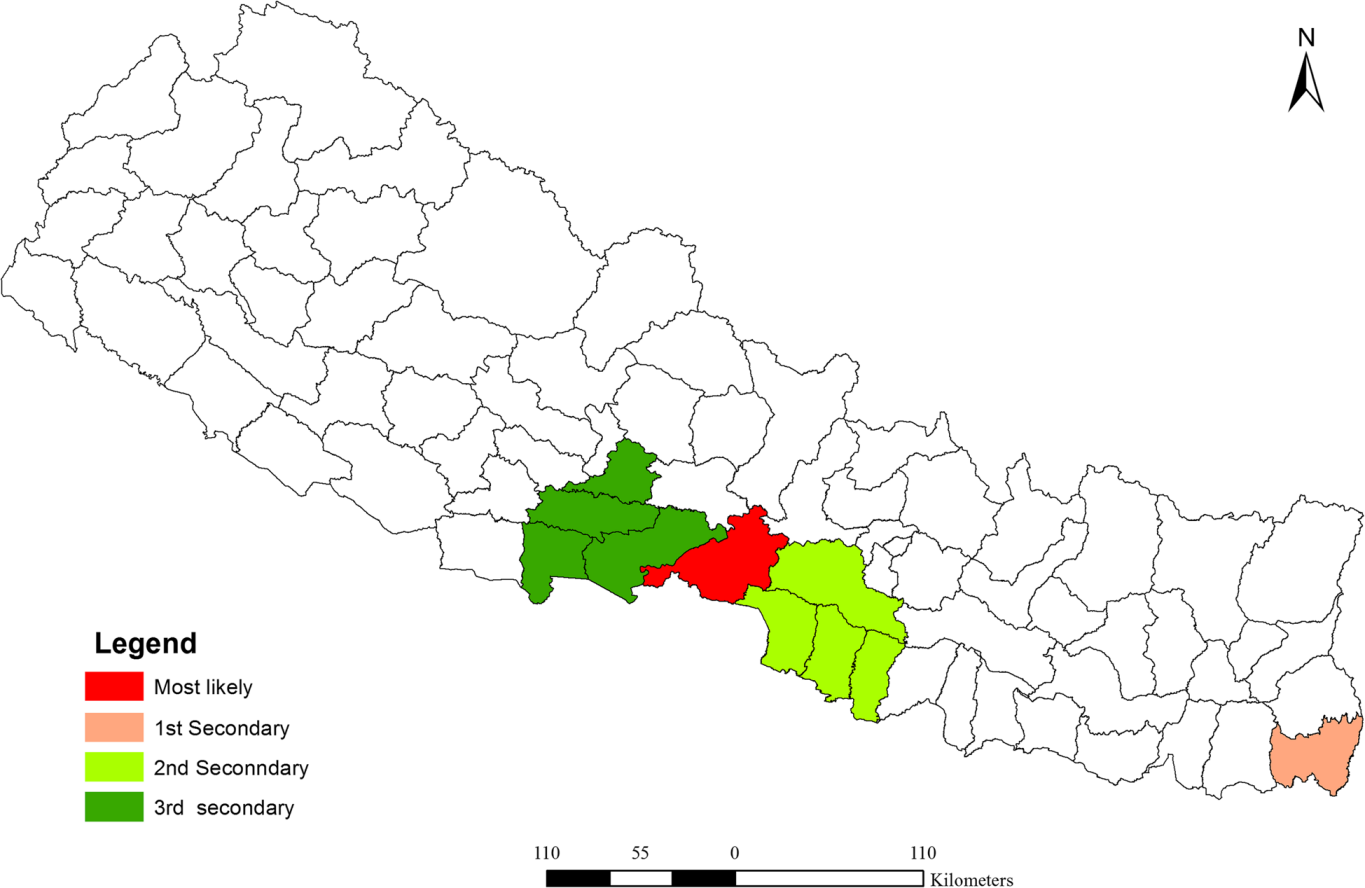
Distribution of spatial clusters of dengue fever in Nepal (2010–2014)

Analysis of purely spatial clustering of dengue cases from 2010 to 2014 produced one of the most likely clusters and three other secondary clusters (Fig. 6). Using the maximum spatial cluster size of 50 % of population at risk, Chitwan district was identified as the most likely cluster and Jhapa district as a first secondary dengue cluster. Bara, Parsa-Makawanpur and Rautahat districts were identified as the second secondary spatial cluster of dengue and Nawalparasi, Rupendehi, Palpa and Syangja districts as the third secondary cluster. Table 1 provides the detailed result of spatial scan analysis.

**Table 1:** Dengue cluster (2010–2014) based on purely spatial analysis under the Poisson Discrete probability model

District	Cluster type	LLR	P	Observed cases	Expected cases	Relative risk
Chitwan	Most likely	3624.83	0.0001	1360	51.31	61.75
Jhapa	1^st^ Secondary	197.17	0.0001	290	71.90	4.46
Parsa, Rautahat, Bara, Makwanpur	2^nd^ Secondary	125.39	0.0001	2298	211.97	1.46
Nawalparasi, Palpa, Rupandehi, Syangja	3^rd^ secondary	31.93	0.00846	239	183.49	1.34

#### Temporal clusters

**Fig. 7:**
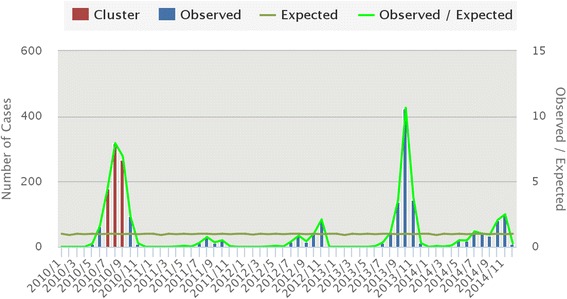
Temporal cluster of dengue fever in Nepal (2010–2014) Spatiotemporal clusters

Purely temporal cluster analysis has identified July to September of 2010 (Fig. 7) to be a strongly significant temporal cluster (Observed = 761, Expected = 118.1, RR = 9.06, LLR = 878.27,7 < 0.001). Observed dengue cases were also significantly high in August 2013 but as temporal scanning method provides single temporal cluster, it was not considered as another temporal cluster in the analysis.

**Fig. 8:**
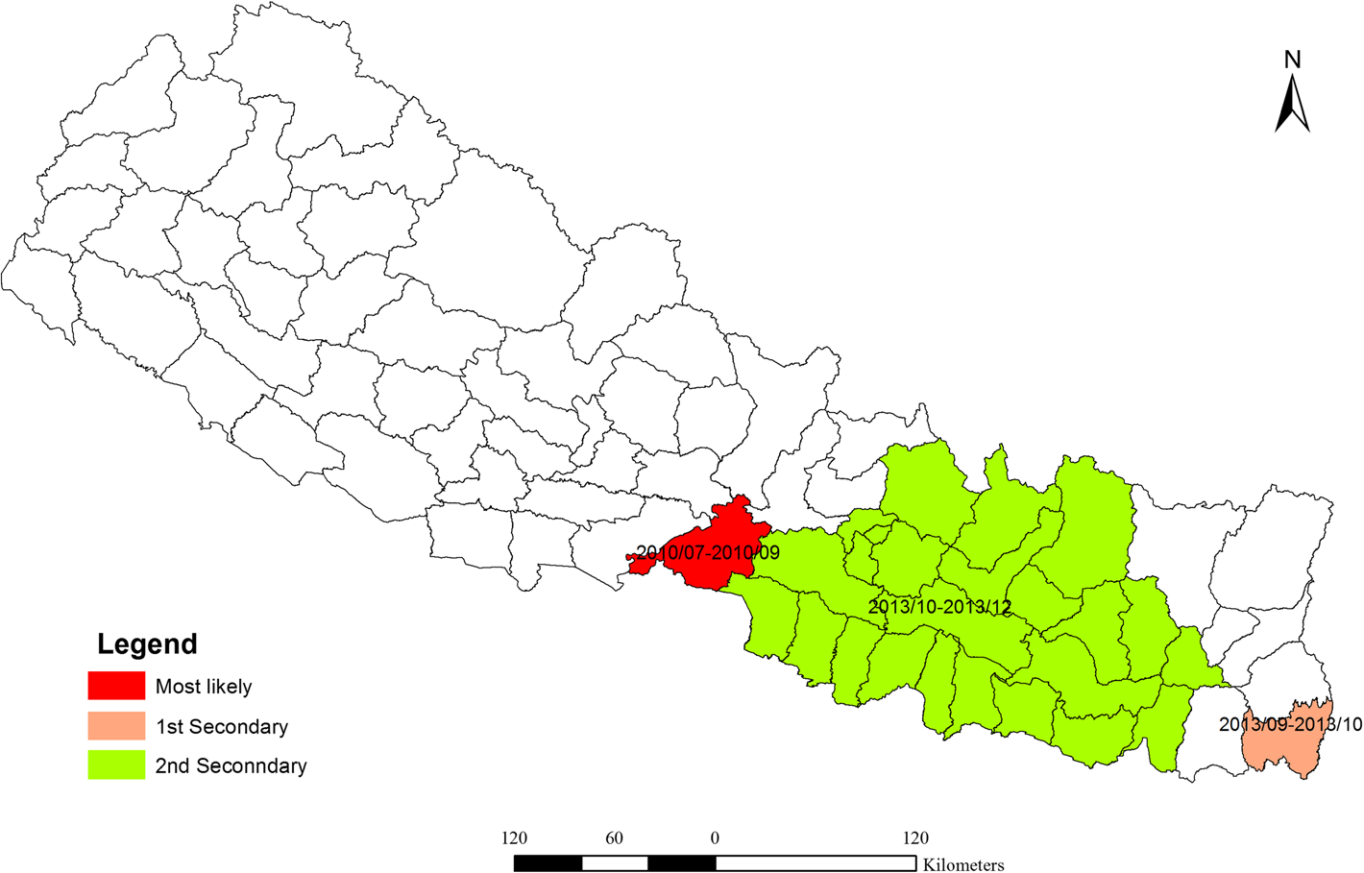
Distribution of spatiotemporal clusters of dengue fever in Nepal (2010–2014)

The space-time cluster analysis of dengue data from 2010 to 2014 was also tested. The result showed (Fig. 8) three non-overlapping statistically significant spatiotemporal clusters. Among them, one was the most likely cluster while the other two were the first and second secondary clusters. The dengue cluster identified from July to September of 2010 at Chitwan district was the most likely cluster. First secondary cluster was identified in Jhapa, from September to October of 2013. The cluster in Parsa and adjoining 23 districts was another secondary cluster from October to December 2013. Table 2 shows the location of each cluster, observed cases, expected cases, relative risk (RR) and log likelihood ratio (LLR).

**Table 2:** Dengue cluster (2010–2014) based on spatial temporal analysis under the Poisson Discrete

Districts	Cluster type	Period	LLR	P	Observed cases	Expected cases	Relative risk
Chitwan	Most likely	2010/7–2010/9	2875.58	0.0001	621	2.59	326.42
Jhapa	1^st^ secondary	2013/9–2013/10	646.12	0.0001	189	2.40	85.51
Parsa + 23 districts	2^nd^ secondary	2013/10–2013/12	97.35	0.0001	102	17.40	6.08

## Discussion

In this study, exploratory data analysis and spatiotemporal cluster analysis of dengue fever were conducted at district level in Nepal. We mapped dengue fever in terms of crude incidence, excess risk and spatially smoothed incidence rate. In addition, we further evaluated spatiotemporal distribution patterns and explored significant spatial, temporal and spatiotemporal clusters. To our knowledge, this is the first attempt to map and analyze spatiotemporal pattern of dengue in Nepal.

Due to availability of data in some sort of spatial aggregation, choropleth mapping technique is popular in disease mapping compared to dot map or isopleths map [33, 38]. Aggregated data is either directly plotted in the map or rate of incidence is computed using base population and level of spatial aggregation. Therefore, we also used choropleth-mapping technique to visualize the distribution of dengue fever. However, when disease incidences or population of area is too small, both the highest and lowest values are concentrated towards the highest values and map becomes misleading [33]. This problem in disease mapping is also known as “small numbers” problem. Small numbers problem commonly appears when the disease is relatively new and not fully endemic across the country or region [39] or due to the variable size of spatial aggregation unit [40]. The advantage of smoothing of geographically aggregated data is that it uncovers unexpected features, patterns and gradients that one might not detect from direct display [41]. In addition, smoothing can reduce unusual values or outliers. Therefore a spatially smoothed map (Fig. 4) presents a better distribution pattern of dengue incidence and shows clearly where the problem was most severe.

The results of the cluster analysis showed the significant spatiotemporal variation of dengue fever in Nepal. Although dengue disease is spreading rapidly to new areas [26, 42], it is highly localized in particular locations and times. Compared to other regions of the country, central and eastern Terai are more vulnerable to dengue. In mapping and cluster assessment, these two districts appeared as a hotspot of dengue. Suitable climate, high population density and excessive movements of the people could attribute to a high dengue cluster. Due to gateway location, we believe that reported dengue cases from other hill districts of this region might have acquired infection from Chitwan. Further investigation is necessary to give more accurate answers about the primary cluster of dengue in Chitwan.

We observed strong inter-annual and seasonal variation of dengue in Nepal. Two major peaks were observed during the 5 years interval: one in August 2010 and another in October 2013 (Fig. 5). Regarding the seasonality, dengue fever follows the pattern of monsoon rainfall. With some time lag, the major outbreak occurs in the post-monsoon seasons; September-November [43]. Post-monsoon season provided the most suitable weather conditions including moderate rainfall and mild mean temperature and optimum temperature range for vector to live [44].

Due to the neighborhood effects [29], we observed an overestimated spatiotemporal cluster including Parsa and its adjacent 23 districts. No dengue incidences were recorded during 2010–2014 in these 23 districts. An overestimated cluster is identified when the expected counts are low and it is surrounded by other location with a lot of cases [35]. Hence careful selection of scanning parameter and interpretation of the result is necessary to better represent and interpret the clusters [45].

Moreover, this study also clearly demonstrated the importance of geospatial technology in spatiotemporal assessments of infectious disease. To our best knowledge, such studies have not been done before in Nepal. Therefore, this study could be an excellent example to promote such studies at higher temporal and spatial scale in the future. Research results and approaches adopted here could be valuable to the public health authority to design and execute an intervention program on dengue control. However, there are some limitations with this study. Possibility of underreporting [23] due to those who did not come to health facilities for treatment and ill cooperation of private health institution in government reporting system is the first limitation of this study. Mapping and analysis on coarsely aggregated data, month and district, may have missed daily or weekly local dengue cluster is the second limitation of this study. If we had daily or weekly dengue cases at lower spatial unit (e.g. settlement, VDC, municipality), we could detect outbreak dynamics and movements of hotspots [29, 37]. Thirdly, this study only analyzed distribution and did not analyze possible environmental risk factors associated with clustering and therefore we could not pinpoint such risk factors. We are expecting to examine such factors in our next research paper.

## Conclusion

This study assessed and mapped the spatiotemporal pattern of dengue fever in Nepal for the first time. Distribution of dengue fever was found highly clustered around Chitwan and Jhapa districts. In the temporal context; dengue is highly seasonal, starts with onset of monsoon, and reaches peak in the post monsoon season. The results of this study are not only to provide an initial risk assessment but also lay foundation to pursue further investigation into the environmental risk factors. This study also clearly demonstrated the importance of geospatial technology in mapping and spatiotemporal assessment of infectious disease. The method adopted here can be used for other diseases and higher spatiotemporal scale. The results of this study may assist health authorities to develop better preventive strategies and increase public interventions effectiveness.

## Abbreviation

DOH, Department of Health Services; EDCD, Epidemiology and Disease Control Division; GIS, Geographic Information System; SaTScan, spatiotemporal scan; VDC, Village Development Committee
